# The Proteomic and Genomic Teratogenicity Elicited by Valproic Acid Is Preventable with Resveratrol and α-Tocopherol

**DOI:** 10.1371/journal.pone.0116534

**Published:** 2014-12-31

**Authors:** Yeh Chen, Ping-Xiao Lin, Chiu-Lan Hsieh, Chiung-Chi Peng, Robert Y. Peng

**Affiliations:** 1 Research Institute of Biotechnology, Hungkuang University, 34 Chung-Chie Rd., Shalu County, Taichung Hsien, Taiwan 43302; 2 Graduate Institute of Biotechnology, Changhua University of Education, 1 Jin-De Rd., Changhua, Taiwan 50007; 3 Graduate Institute of Clinical Medicine, College of Medicine, Taipei Medical University, 250 Wu-Shing St., Taipei, Taiwan 11031; 4 Research Institute of Medicinal Sciences, College of Medicine, Taipei Medical University, 250 Wu-Xing St., Taipei, Taiwan 11031; Second University of Naples, Italy

## Abstract

**Background:**

Previously, we reported that valproic acid (VPA), a common antiepileptic drug and a potent teratogenic, dowregulates RBP4 in chicken embryo model (CEM) when induced by VPA. Whether such teratogenicity is associated with more advanced proteomic and genomic alterations, we further performed this present study.

**Methodology/Principal Findings:**

VPA (60 µM) was applied to 36 chicken embryos at HH stage 10 (day-1.5). Resveratrol (RV) and vitamin E (vit E) (each at 0.2 and 2.0 µM) were applied simultaneously to explore the alleviation effect. The proteins in the cervical muscles of the day-1 chicks were analyzed using 2D-electrophoresis and LC/MS/MS. While the genomics associated with each specific protein alteration was examined with RT-PCR and qPCR. At earlier embryonic stage, VPA downregulated *PEBP1* and *BHMT genes* and at the same time upregulated *MYL1, ALB* and *FLNC* genes significantly (*p*<0.05) without affecting *PKM2* gene. Alternatively, VPA directly inhibited the folate-independent (or the betaine-dependent) remethylation pathway. These features were effectively alleviated by RV and vit E.

**Conclusions:**

VPA alters the expression of *PEBP1, BHMT*, *MYL1, ALB* and *FLNC* that are closely related with metabolic myopathies, myogenesis, albumin gene expression, and haemolytic anemia. On the other hand, VPA directly inhibits the betaine-dependent remethylation pathway. Taken together, VPA elicits hemorrhagic myoliposis via these action mechanisms, and RV and vit E are effective for alleviation of such adverse effects.

## Introduction

Valproic acid (VPA, 2-propylpentanoic acid)) is a short-chain fatty acid commonly used as an anticonvulsant [1] in the long-term treatment of epilepsy [2]–[5], which has been recently identified as a histone deacetylase (HDAC) inhibitor, leading to the acetylation of histone tails and regulation of gene expression [6], [7]. VPA modulates the status of epilepsy via inhibiting citric acid cycle and elevating γ-aminobutyric acid levels in the central nervous system [2]. Recently, we have reported that VPA induced a diversity of teratogenic features in chicken embryo model (CEM) [4], [8], [9]. VPA downregulated levels of superoxide dismutase (SOD), glutathione (GSH), HDAC and folate; while VPA upregulated H_2_O_2_ and homocysteine [3]. VPA dowregulates retinol binding protein 4 (RBP4) [4]. The microarray analysis revealed 17 genes downregulated and four upregulated [5]. The relevancy covered translation (23%), signal transduction (23%), transcription (16%), cell adhesion (16%), neural cell migration (8%), transport (7%), and organismal development (7%). VPA downregulated several genes like insulin-like growth factor 2 receptor (*IGF2R*), regulator of G-protein signaling 4 (*RGS4*), alpha 3 (VI) collagen (COL6A3), endothelin receptor type b (*EDNRB*), Krüppel-like factor 6 (*KLF6*) and folate receptor 1 (*folr1*), which are directly associated with neural tube defect (NTD) [5]. VPA induces haemorrhagic liposis of the cervical muscles in the chicken embryo model (CEM) [6].

Pharmacologically VPA elicits teratogenicity at three levels, i.e. the genomic, the proteomic and the metabolomic levels. At the genomic level, VPA inhibits HDAC gene [8], [10], [11]; compromises DNA by reducing folate supply [12] and directly damages DNA [13].

Histone acetylation modulates transcription in multiple ways. Its enzymes, acetyltransferases, and deacetylases can regulate transcription by modifying the acetylation state of histones or other promoter-bound transcription factors

Histone acetylation effectively reduces the positive charge of histones, and this has the potential to disrupt electrostatic interactions between histones and DNA [14]. This presumably leads to less compact chromatin structure, thereby facilitating access of DNA to molecular machineries involved in transcriptional control. Conversely, histone deacetylation favors transcriptional repression by inducing chromatin compaction [15].

Menegola et al. indicates that VPA exerts its teratogenic effect by inhibiting histone deacetylases and by binding to the retinoic acid (RA) receptor [10]. At 24 hpf, exposure to 320 µM VPA induced increased Aldh1a2 (aldehyde dehydrogenase 1 family, member A2) expression in the somites and decreased expression in the branchialarches [16].

Gene expression profiling of multiple myeloma (MM) cells exposed to VPA showed downregulation of genes involved in cell cycle progression, DNA replication and transcription, as well as upregulation of genes implicated in apoptosis and chemokine pathways [17].

Similarly to the Aldh1a2 pattern, the expression pattern of cytochrome P450, family 26, subfamily A, polypeptide 1 (Cyp26a1) in embryos exposed to VPA at 24 hpf also showed evidence of up-regulation. In older embryos/larvae (≥48 hpf), the expression pattern of Cyp26a1 in the group exposed to VPA was similarly downregulated as the control group [16].

At the proteomic level, VPA elicited folic acid deficiency via retarding methylene tetrahydrofolate reductase (MTHFR) [3], [4], [18]–[20]. In addition, VPA also inhibited SOD, HDAC and RBP4 etc [3], [4]. Mild MTHFR deficiency and reduced maternal erythrocyte folate concentration are considered to be strong risk factors for NTD [21]. In CEM, VPA substantially altered the levels of RBP4, protein SET, apolipoprotein A-1, carbonic anhydrase 2, NADH-cytochrome b5 reductase, 60S ribosomal protein 122 (ovotransferrin), and downregulated superoxide dismutase [5]. While at metabolomic level, VAP reduced serum folate, leading to hyperhomocysteinemia that has been considered a mediator of the teratogenic potential of VPA [5], [18]. VPA downregulated glutathione (GSH) and upregulated the serum levels of H_2_O_2_ to provoke severe oxidative stress [5], [22], which in turn triggered β- and ω-oxidations [23]. In addition, VPA actively induces antiangiogenesis [24], hepatotoxicity, hepatic steatosis, and alteration of mitochondrial function [25]. More recently, in day-1 chicks we found VPA uniquely induced muscular hemorrhagic liposis in the cervical muscles when administered at early embryonic stage (HH stage 10, day-1.5 embryos) [9]. The major cause was shown closely associated with the simultaneous upregulation of acetyl CoA carboxylase (*ACC*) and downregulation of carnitine palmitoyl transferase-1 (*CPT1*) genes in the HH stage 22 (day 3.5) CEM [9]. However, the detail action mechanism of VPA playing the role at the genomic and proteomic levels in CEM to induce hemorrhagic myoliposis until hatched still remains unclear. In the meanwhile, whether the alterations of gene expression affected by VPA could be prevented by nutraceutics like resveratrol and vitamin E, we carried out this present investigation.

## Methods

### Animals

The whole animal experiment project has been approved by the National Changhua University of Education Ethic Committee with the Guide for the Care and Use of Laboratory Animals as adopted and promulgated by the U.S. National Institutes of Health. Previously, we showed that the VPA at a tissue level 60 µmolL^−1^ (i.e. 100 µL of 30 molL^−1^ VPA per egg to give a final tissue level 60 µmolL^−1^) yielded an optimum malformation rate 36.5±3.0% with a mortality rate 47.7±0.5% [4], hence this single level dose 60 µmolL^−1^ was adopted throughout this experiment. Unless otherwise stated, the protocol conducted was similar to the previously cited [4]. Briefly, fertilized eggs purchased from the Haw-Yang Agricultural Farm (Taichung, Taiwan) were divided into 10 groups, each 36 eggs. These eggs were incubated at 37°C and RH 70–80% until HH stage-10 (day 1.5 embryo). Different treatments were applied as follows: group 1, the normal control; group 2, the VPA (60 µM)-control; groups 3 and 4, the Resveratrol (RV) 0.2 µm and 2,0 µM controls; groups 5 and 6, VPA 60 µM plus RV 0.2 µM and VPA 60 µM plus RV 2.0 µM; groups 7 and 8, the vitamin E (vitE) 0.2 µM and 2,0 µM controls; groups 9 and 10, VPA 60 µM plus vit E 0.2 µM and VPA 60 µM plus vitE 2.0 µM, respectively. The incubation was continued until the HH stage 46 (day-1) chicks were subjected to CO_2_-euthanesia [26]. The cervical muscles were excised and rinsed twice with 4°C PBS buffer. Part of the tissue was frozen immediately for Hematoxylin-Eosin staining and the remaining muscle samples were homogenized with 4°C PBS on ice (EMH).

### Protein extraction

Protein extraction was conducted as previously reported [27]. In brief, 500 µL of lysis buffer mixture (each 10 mL lysis buffer + protease inhibiter 100 µL) was added to 100 mg of EMH. The mixture was gently agitated on the ice rock for 10 min to facilitate the reaction and then homogenized at 4°C and centrifuged at 14000 g and 4°C for 20 min. The supernatant mixture containing the intracellular protein was stored at −80°C for use (the protein extract, PrE). The extraction for protein was repeated at least with six replicates.

### 2D-gel electrophoresis

#### 2D-clean up

PrE (3 mg) was transferred to a 1.5 mL tube, to which 300 µL precipitant was added. The mixture was agitated for 4–5 s and left to stand at 4°C for 15 min. Co-precipitant (300 µL) was added and agitated. The mixture was centrifuged at 8000 g at 4°C for 10 min. The supernatant was carefully removed. The residual pellet was re-centrifuged at 8000 g at 4°C for 5 min to remove remaining supernatant. To the pellet 40 µL of double distilled water was added. The proteins was dispersed using a thin spatula and then ultrasonicated for 1 min. Wash buffer (1 mL), previously cooled to −20°C, was introduced with 5 µL of additive solution to the mixture and agitated at −20°C at 10 min intervals for 30 min. The solution was left to stand at −20°C for 30 min and centrifuged at 8000 g at 4°C for 10 min. The supernatant was carefully removed. The pellet was nitrogen gas blown to dry for 5 min. The residue was redissolved at ambient temperature in 420 µL rehydration buffer containing DDT. The solution was left to stand for 30 min and centrifuged at 8000 g for 10 min to remove any insoluble particles. An aliquot of the supernatant (350 µL) was used to carry out the IEF electrophoresis.

#### IEF electrophoretic analysis

The strip holder was first rinsed with neutral detergent to remove the protein residues left from the previous work, then washed twice with double distilled water and left to dry at ambient temperature. The protein sample (3 mg) prepared as described in the above was spread evenly onto the strip holder (Hoefer TM TE22). Two filter pads after having previously been wetted with double distilled water were placed onto the two electric terminals on the focusing tray. The protective covering membrane on the IPG strips (immobilized pH gradient strip) was removed. The IPG strips were fixed face down on to the focusing strip. Mineral oil (2.5 mL. IPG Cover Fluid) was placed onto the IPG strip, avoiding the evaporation of the rehydration buffer. The whole focusing tray was placed onto a Protean IEF cell (Bio-Rad Laboratories Inc.) (California, USA) and electrophoresis was conducted at 20°C. The subsequent protocols involved rehydration at 50 V for 12 h and conditioning at 250 V for 15 min, followed by raising the electric potential up to the focusing voltage. During the IEF electrophoresis, the 7-cm long IPG strips were focused at a voltage of 4000 V for a V*h of 20000 V*h. For IPG of length 11 cm and 17 cm, the corresponding values should be set at 8000 V and 40000 V*h, and 10000 V and 6000–8000 V*h, respectively. After focusing, the voltage was set at 500 V to avoid further diffusion of the proteins already focused. For each sample, six replicates of electrophoretic analysis were performed.

#### SDS-PAGE

After IEF electrophoresis, the IPG strips were rinsed with double distilled water to remove the residual mineral oil. Fresh balancing fluid (containing DDT) was added and the solution was left to stand for 20 min to allow equilibration. Then the fluid was removed and more balancing fluid (containing IAA) was added and the strips were left to be equilibrated for 20 min. The equilibrated IPG strips were then placed onto a 10% SDS-PAGE gel and tightly contacted. Then 0.5% agarose solution was used to cover and fix the strips. The gel was placed in either a MiniProtean II cell or a Protean II xi 2-D cell. After the protein marker (5 µL) had been added, the electrophoresis was started using the conditions: 4°C, 16 mA/gel for 30 min, and then 35 mA/gel for up to 5 h until the dye had reached the bottom of the gel. For each 2D analysis, at least six replicates were performed.

#### Silver staining

The SDS-PAGE was removed from the apparatus and the silver stain was carried out as follows: i) fixed for 30 min with a fixation agent (95% ethanol 210 mL, acetic acid 50 mL, and double deionized water to make up to 1000 mL); ii) sensitized for 30 min with an sensitizing solution (95% ethanol 158 mL, sodium thiosulfate 2.44 g, sodium acetate 34 g, and double deionized water to make up to 500 mL; iii) rinsed with double deionized water six times, each time for 5 min; iv) silver stained for 20 min with staining solution (500 mL of 0.25% silver nitrate solution containing 200 mL of 37% formaldehyde); v) rinsed twice with double deionized water, each time for 1 min; vi) developed with developing solution (sodium carbonate 12.5 g, 100 mL of 37% formaldehyde, and double distilled water (ddw) to make up to 1 L); and vi) halting the reaction with stopping fluid (acetic acid 25 mL and ddw 475 mL). The developed SDSPAGE was scanned using Melanie 7 software to pinpoint protein spots that were significantly different (*p*<0.05).

### Identification of proteins by LC-MS/MS

#### MS sample preparation

Spots on the SDS-PAGE was rinsed twice with mine Q water wash, each time for 10 min. The spots excised from the stained gels were processed according to the standard MS preparation protocol [28], [29]. In-gel digestion of proteins was carried out using MS-grade Trypsin Gold (Promega, Madison, WI) overnight at 37°C. Tryptic digests were extracted using 10 µL Milli-Q water initially, followed by two times of extraction with a total of 20 µL mixed solvent (containing acetonitrile/trifluoroacetic acid  = 50%/0.1%). The combined extracts were dried in a vacuum concentrator at ambient temperature. The desiccated residue was dissolved in 1 µL mixed solvent containing 5% acetonitrile/0.5% trifluoroacetic acid.

#### EIS-MS/MS analysis and protein identification

The EIS-MS/MS mass spectrometer Thermo LTQ Orbitrap (Thermo Scientific, UK) was utilized for protein analysis. The MS/MS signal analyzed by using the MASCOT searching engine (www.matrixscience.com). The search parameters were defined as follows: Database, NCBInr 20130609; Taxonomy, Other lobe-finned fish and tetrapod clade; Peptide MS tolerance, ±0.5 Da; Fragment MS tolerance, ±0.5 Da and allowance of one missed cleavage.

Before each sample analysis, 50 fmol of tryptic BSA standard was used to confirm the column efficiency and EIS-MS/MS sensitivity. For the MASCOT search, the data was deisotoped and converted by the Data Analysis Version 4.0 (Build 275).

The EIS-MS/MS analysis was repeated for at least six replicates. Peptides were considered as identified if their MASCOT individual ion score was higher than the MASCOT score 30 (*p*<0.001).

### PCR for quantification of genes ACC (acetyl CoA carboxylase) and CPT-1

#### Extraction of RNA

The protocol used for extraction of RNA was carried out according to the manufacturer's instruction (Sigma; Poole, England). To 100 mg of pecking muscle tissue obtained from day-1 (HH-stage 46) chicks, 1 mL of TriReagent (Sigma; Poole, England) was added and homogenized. And the remaining procedures were conducted as instructed (Sigma; Poole, England). The content of RNA was assayed with Nono-Drop 1000 Spectrophotometer (Thermo Fisher Scientific Inc., Waltham, MA, USA) by calculating the ratio OD_260_/OD_280_ (standard grading should reach A_260_/A_280_ = 1.7–1.9). The extraction of RNA was repeated for at least six replicates to assure the accuracy. The purified RNA obtained was assayed and stored at −80°C for use. The experiments were repeated for six times.

#### RT-PCR

The RNA sample was carefully measured and the RT-PCR was carried out to produce the c-DNA according the manufacturer's instruction (Genemark, Taiwan containing MMLV reverse transcriptase). The RT-PCR was repeated for at least six replicates to assure the accuracy.

#### qPCR

The cDNA obtained was multiplied with qPCR MJMini (Bio-Rad, USA) kits using the primers with nucleotide sequence indicated below. The primers of the selected target genes are listed in Table 1. The amount of c-DNA obtained was assayed with GelPro31 as previously described [9]. The OD at 260 nm and 280 nm were taken. The ratio A_260_/A_280_ = 1.7–2.0 was considered to be optimum. The product obtained was stored at −20°C for use.

**Table 1:** Primer sequences used in real time PCR and RT-PCR.

Gene name	Primers position	Primers sequence (5′→3′)	Product size (bp)
PEBP1	Forward	GACAAACGAGGGAAGTTCA	201
	Reverse	CAGTGCTTGGTCAGGTC	
MYL1	Forward	CTCCAAACCCACTACTTTCT	188
	Reverse	AACAGGGATTTACTGATGCT	
ALB	Forward	CTCCGTGACTCTTATGGTG	168
	Reverse	TCCTGGTATTCCTGGCA	
BHMT	Forward	CTTGTGACATTGCCAGAGA	250
	Reverse	TTTAGGACTTCAACTGCCCA	
PKM2	Forward	ACTGAGGTTGAGAACGG	450
	Reverse	CGGATGAAGGAAGCGAACA	
FLNC	Forward	CGACAAGGTGAAGGTTTGG	1125
	Reverse	GACGATGACGGTGTGTT	

#### Agarose gel electrophoresis

The required amount of Agarose Ultra Grade for molecular biology (Agarose Type II, Sigma Co., USA) depends upon the molecular size of DNA fragments to be analyzed. In this experiment, 2% was used. Briefly, Agarose Ultra Grade was accurately measured and dissolved in 5 fold-diluted TAE buffer with the aid of a microwave heater. The dissolved mixture appeared to be transparent without any contamination. To each 100 mL the agarose solution 3 µL of safe view DNA stain internal dyeing agent was added and left to cool down at ambient temperature for 10 min. The cooled mixture was poured into the casting mold. The air bubbles were carefully driven off and the cool-down process was continued at ambient temperature avoiding direct sunlight until completely solidified. The finished Agarose mixture was placed into the electrophoresis chamber. TAE buffer (×5) was used as the electrophoresis buffer solution. The required amount of c-DNA (approximately 100 ng/µL) was carefully measured, thoroughly mixed with 6-fold diluted loading dye, and injected into the SDS-PAGE chamber concave. A 100-volt potential was applied for 20 min and the image was taken with the Luminescence/UV Image System. The sample band was counter-viewed with DNA ladder to ensure the light band width to be as large as expected and the photos were taken for quantification. The agarose gel electrophoresis was repeated for at least six replicates to assure the accuracy.

#### Data analysis

Data were treated with ANOVA and a post hoc test using SigmaPlot 8.0 and Systat 10.0 (SPSS Inc., Chigago, III). Comparison of 2 means was performed using paired or unpaired Student's *t* test, as appropriate. All *p* values were two-tailed, and *p*<0.05 was required to reject the null hypothesis. Results were expressed as mean±SEM of triplet experiments.

## Results

### The malformation and mortality rates occurred in the embryos

The malformation- and mortality rates caused by valproic acid (60 µM) and the alleviative effect of resveratrol and vitamin E is shown in Table 2.

**Table 2:** The malformation- and mortality rates caused by valproic acid and the alleviative effect of resveratrol and vitamin E.*

group	Incidence, %		
	normal	malformation	mortality
control	91.5±6.0^a^	0	8.5±6.0^c^
VPA 60 µM	15.8±2.3^d^	36.5±2.5^a^	47.7±0.5^a^
RV 0.2 µM	91.5±2.0^a^	0	8.5±6.2^c^
RV 2.0 µM	94.8±3.1^a^	0	5.0±4.2^c^
VPA+RV 0.2 µM	70.7±6.2^c^	12.0±1.5^b^	17.4±6.2^b^
VPA+RV 2.0 µM	86.2±0.7^b^	8.4±1.5^c^	5.5±0.7^c^
Vit E 0.2 µM	90.0±10.0^a^	0	10.0±7.0^c^
Vit E 2.0 µM	86.7±5.8^b^	0	13.3±5.8^c^
VPA+Vit E 0.2 µM	75.0±13.2^b^	8.3±2.9^c^	16.7±12.6^b^
VPA+Vit E 2.0 µM	90.0±4.5^a^	5.0±1.0^d^	5.0±3.0^c^

*Data expressed as mean ± SD (n = 12/36). Data in the same column with different superscripts in lower case indicate significantly different from each other among the different treatment groups (*p*<0.05).

VPA at 60 µM induced a malformation rate and a mortality rate of 36.5±2.5 and 47.7±0.5%, respectively, compared to 0.0 and 8.5±2.5% for the control (*p*<0.05) (Table 2). The RV and vit E controls all showed comparable results. On treatment with RV (0.2 µM and 2.0 µM) and vitE (0.2 µM and 2.0 µM), the malformation rates were reduced to below 12.0±1.5% by RV and to below 8.3±2.9% by vitE. The corresponding mortality rates were suppressed to below 17.4±6.2% and 16.7±12.6%, respectively (*p*<0.05) (Table 2). Higher dose of RV and vitE seemed to be more beneficial to the alleviation.

### VPA induced hemorrahgic myoliposis in cervical muscles

**Figure 1:**
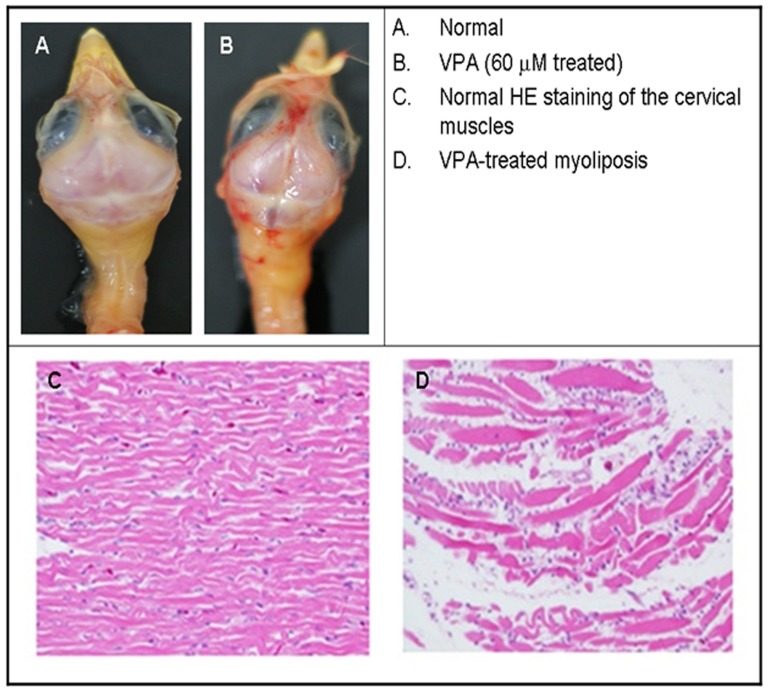
Hemorrhagic myoliposis of cervical muscles induced by VPA. Cervical musclular myoliposis (A, normal vs. B, malformed). Histopathological examination (C, normal vs. D, malformed) (magnification, 400×). Fig. D shows the completely destruction of myofibril lineages by VPA (60 µM) (n = 36).

VPA (60 µM) administered at early stage of embryonic development caused severe hemorrhagic myoliposis (Fig. 1A vs. 1B). The prevalence rate reached 36.5±3.0% [9]. Due to hemorrhagic myoliposis, the cervical muscles were severely damaged, resulting in disabled pecking ability. Hematoxylin-Eosin staining revealed distinctly the myofibre lineage was entirely destroyed by liposis occurring in the cervical muscular tissues (Fig. 1C vs. 1D).

### Protein expressions affected by different treatments

**Figure 2:**
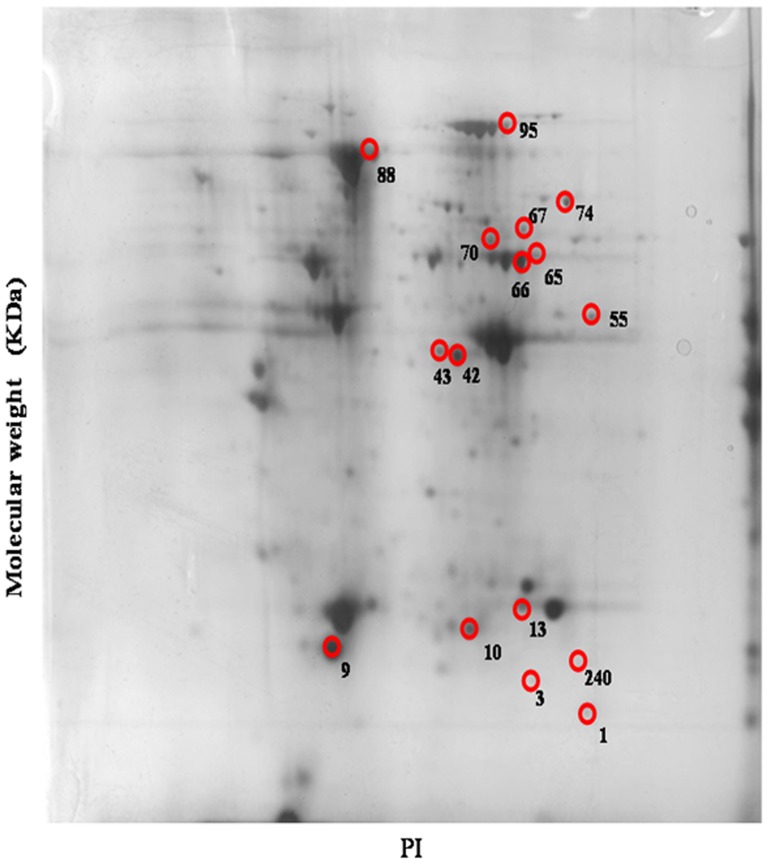
Protein analysis by 2D-gel electrophoresis. Sixteen proteins in the cervical muscle of day-1 chicks were significantly altered by VPA (60 µM) treatment. Six replicates were carried out to assure the results.

**Figure 3:**
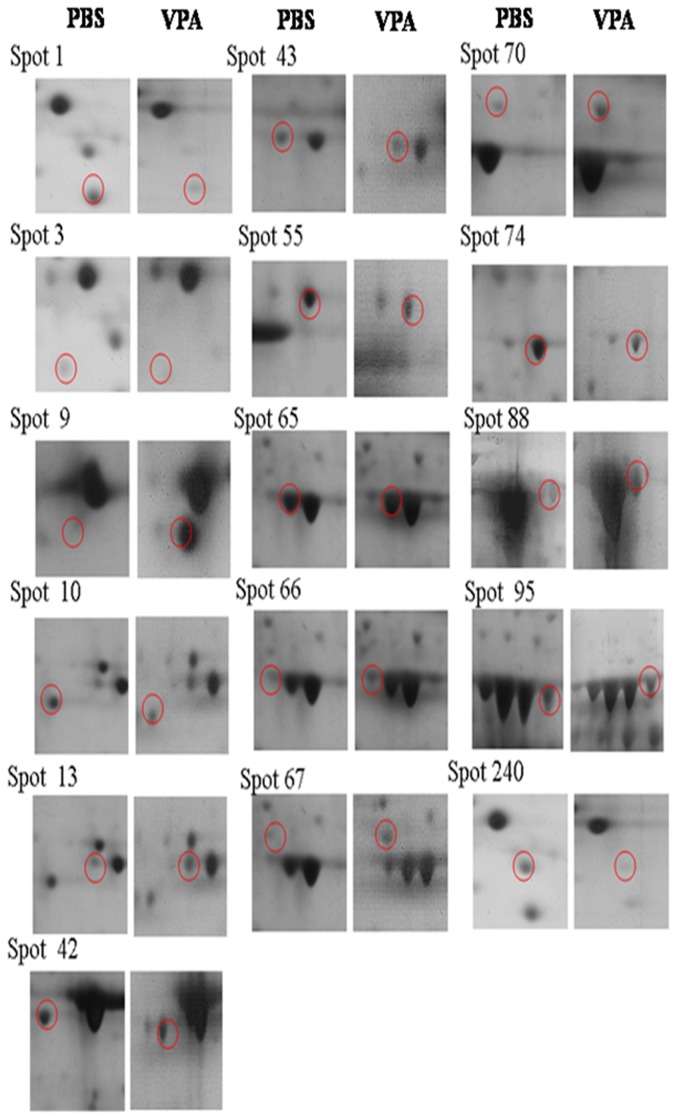
Comparison between the normal and the altered protein spots that were significantly affected by VPA. The phosphotidylethanolamine–binding protein (PEBP1), myosin light chain 1 (MYL1), triose phosphate isomerase (TIM), filamin C (FLNC), pyruvate kinase muscle isozyme (PKM), serum albumin precursor (ABL), and peroxiredoxin-1 (PRDX1) showing a fold change >1.8 were further treated with RT-PCR and qPCR (referred to S1 Table). The dose of VPA used was 60 µM.

LC/MS/MS revealed 16 protein spots on 2D-electrophoresis had been substantially altered due to administration of VPA at early embryonic stage (HH stage 10, day 1.5 embryos). These spots were denoted No. 1, 3, 9, 10, 13, 42, 43, 55, 65, 66, 67, 70, 74, 88, 95, and 240 (Fig. 2), respectively, and their intensity was compared with that of the PBS treated control (Fig. 3).

The proteins expressed in chicken cervical muscle samples were identified with LC/MS/MS (see S1 Table). The analysis was repeated for six replicates. Preliminarily, spots having significant difference at a CL level *p*<0.05 were collected (Table 3). The theoretical molecular weight and their isoelectric point are shown in Table 3. From the assigned proteins the mostly relevant proteins were selected based on the fold changes (FC) ≥1.8 (Table 3). Which included (Number, FC, gene) phosphatidylethanolamine binding protein 1 (No. 1, FC = −2.0816, *PEBP1*); myosin light chain 1 (No. 9, 2.9896, *MYL1*); triose phosphate isomerase (No. 13, 2.6785, *TIM*); betaine-homocysteine S-methyltransferase (No. 55, −1.2048, BHMT); filamin C (No. 67, 2.4397, *FLNC*); pyruvate kinase muscle isozyme (No. 74, −1.8109, *PKM*); serum albumin precursor (No. 88, 3.7126, *ALB*); and peroxiredoxin 1 (No. 240, −2.1684, *PRDX1*) (Table 3).

**Table 3:** Summary of the 16 protein spots identified at significant CL *p*<0.05.^a^

SSP	Protein name	Gene	Fold changes (VPA/PBS)	P-value	MOWSE score	Accession No.	Coverage [%]	Theoretical Mr[Da]/pI
1	Phsphatidylethanolamine-binding protein 1	PEBP1	−2.0816	0.01641	396	gi|310772215	56%	21.12/6.96
3	Ovalbumin	OVA	−1.4683	0.02587	334	gi|129293	24%	43.20/5.19
9	Myosin light chain 1	MYL1	2.9896	0.02331	965	gi|55584149	64%	20.94/4.96
10	Heat shock protein beta-1	HSPb1	−1.2180	0.00456	453	gi|45384222	57%	21.72/5.77
13	Triose phosphate isomerase	TIM	2.6785	0.03322	837	gi|230359	71%	26.76/7.26
42	Fructose-bisphosphate aldolase C	ALDOC	1.5592	0.04458	1555	gi|330417943	68%	39.74/6.20
43	Fructose-bisphosphate aldolase C	ALDOC	1.4391	0.04458	1166	gi|330417943	55%	39.74/6.20
55	Betaine—homocysteine S-methyltransferase	BHMT	−1.2048	0.02658	1764	gi|507552288	76%	45.55/7.56
65	Beta-enolase	ENO-3	1.6320	0.00987	705	gi|46048765	45%	47.57/7.28
66	Beta-enolase	ENO-3	1.1248	0.03641	475	gi|46048765	43%	47.57/7.28
67	Filamin-C	FLNC	2.4397	0.04591	465	gi|45383033	10%	283.23/5.93
70	Dihydrolipoyl dehydrogenase	DLD	1.6761	0.02639	130	gi|71897021	6%	54.57/8.19
74	Pyruvate kinase muscle isoenzyme	PKM	−1.8109	0.04851	2934	gi|45382651	81%	58.43/7.29
88	Serum albumin precursor	ALB	3.7126	0.04981	937	gi|45383974	58%	71.87/5.51
95	Ovotransferrin	LTF	−1.2363	0.01874	1818	gi|71274079	79%	77.52/6.70
240	Peroxiredoxin-1	PRDX1	−2.1684	0.04688	254	gi|429836849	42%	22.53/8.24

(n = 36). ^a^Data were treated with ANOVA and a post hoc test using SigmaPlot 8.0 and Systat 10.0 (SPSS Inc., Chigago, III). Comparison of 2 means was performed using paired or unpaired Student's *t* test, as appropriate. All *p* values were two-tailed, and *p*<0.05 was required to reject the null hypothesis. Results were expressed as mean±SEM of triplet experiments.

### Gene expressions affected by different treatments

**Figure 4:**
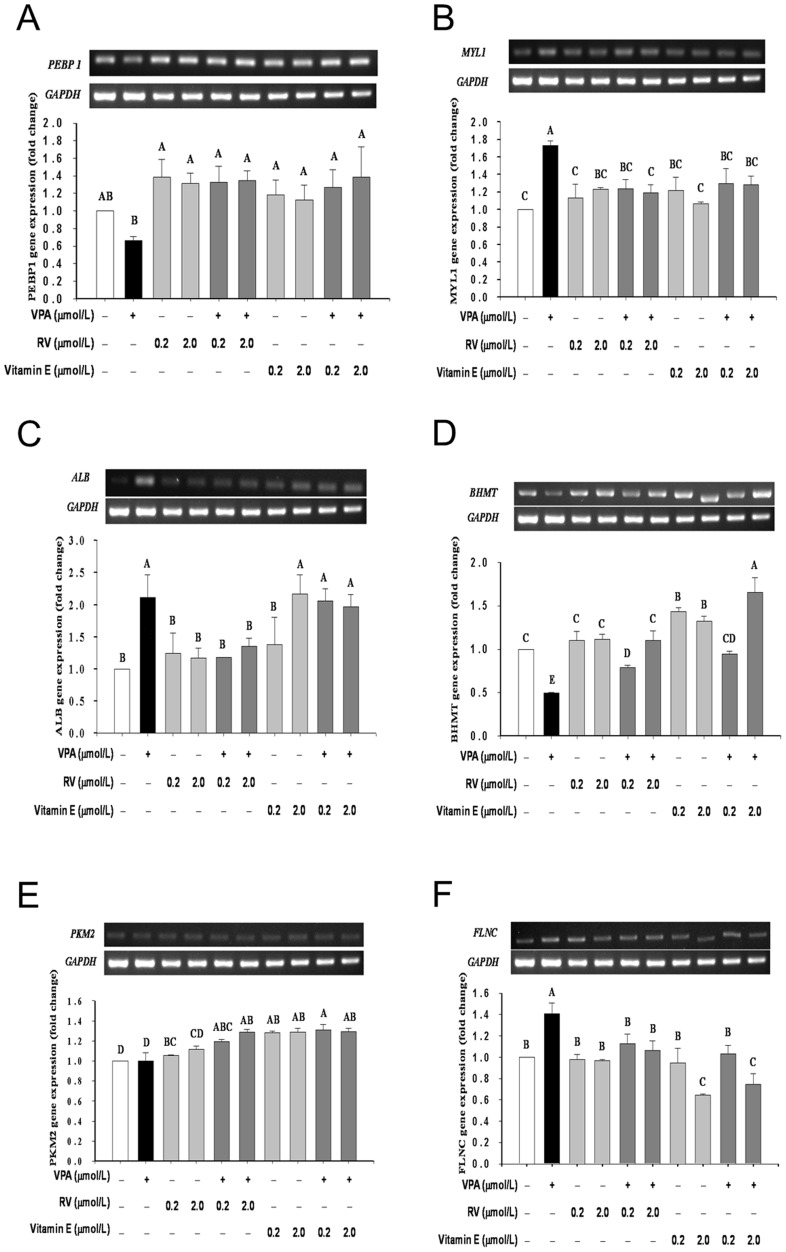
RT-PCR and qPCR showed six gene expressions were significantly altered after treated with VPA. Kits for identification of triose phosphate isomerase (TIM) and peroxiredoxin-1 (PRDX1) genes were then still lacking. Alternatively, we selected betaine-homocysteine S methyl transferase (BMHT) (having a fold change  = −1.2048) to make up 6 genes in this experiment. Experiment was repeated for six times to assure the results. Data are expressed in Mean±SD (n = 36). The dose of VPA used was 60 µM.

Considering the cited pharmacological effect of VPA [5], [8], [10], [11], [21], we further carried out the RT-PCR with subsequent qPCR to determine the expression of these selected genes *PEBP1, MYL1, BHMT, PKM, FLNC* and *ALB*. The expression affected by VPA, RV and Vit E is shown in Figures 4A-4F. Densitometric analysis revealed VPA at 60 µM downregulated genes *PEBP1* and *BHMT* to 0.7 and 0.5 folds respectively (*p*<0.05) (Fig. 4A, 4D). RV and Vit E alleviated the downregulated *PEBP1* despite of the dose use (*p*<0.05). As contrast, RV and Vit E ameliorated the downregulated *BHMT* in a dose-dependent manner (*p*<0.05). On the other hand, VPA upregulated genes *MYL1*, *ALB* and *FLNC* (*p*<0.05) (Figures 4B, 4C, 4F). Both RV and Vit E effectively restored level of gene *MYL1* against upregulation by VPA (*p*<0.05) (Fig. 4B). Although *PKM2* was totally unaffected by VPA, both RV and Vit E substantially elevated *PKM2* even in the presence of VPA (*p*<0.05) (Fig. 4E). On the other hand, VPA therapy significantly upregulated gene *FLNC* (*p*<0.05) (Figure 4F) which was alleviated by RV and Vit E in a dose dependent manner (*p*<0.05) (Fig. 4F).

## Discussion

### The cause of malformation and mortality induced by VPA

Previously we reported that the cause eliciting malformation and mortality by VPA involved multi-mechanism, such as inhibition of histone deacetylase (HDAC), suppressed superoxide dismutase (SOD) and glutathione regenerative cycle; enhanced ROS stress, and neural tube defect (NTD) [3]; [5], [6], downregulated gene folr1, IGF2R, RGS2, COL6A3, EDNRB, KLF6, and pax-3 [5]. VPA inhibited methionine biosynthesis by inhibiting the methylation of homocysteine. VPA downregulated cervical muscular carnitine, *CPT1* expression and GSH, and at the same time, increased levels of triglycerides, H_2_O_2_, and malondialdehyde (*p*<0.05) [6].

### Role of gene phosphotidylethanolamine binding protein 1 (PEBP1)

PEBP protein (synonymous Raf Kinase Inhibitory Protein, RKIP) inhibits the Raf/MEK/ERK cascade, appearing to support macrophage differentiation via inhibition of the NFKB pathway and acting as a novel effector of apoptosis signaling [30], [31]. Gene *RKIP* is expressed in prostate epithelium, brain, liver, lung, testis, muscles and stomach [32], [33]. While RKIP is localized in the cytoplasm and at the plasma membrane spermatids, Leydig cells, oviduct and ovary, mammary glands, uterus, thyroid, steroidogenic cells of the adrenal gland zona fasiculata, small intestine, plasma cells, Schwann cells, and Pukinje cells and stomach (Atlas of Genetics and Cytogenetics in Oncology and Haematology). Loss of RKIP induces radioresistance in prostate cancer [34]. While the documented direct effect of VPA on gene *PEBP1* (*RKIP*) is still lacking, to our believe we are the first who show VPA downregulated *PEBP1* (*RKIP*) at the early embryonic stage, implicating a diverse wide-range, if not a systematic, deficits associated with VPA therapy. RV and Vit E completely rescued such deficits (Fig. 4A).

### Role of gene MYL1 (MLC1, myosin light chain 1)

Molecular and cellular modifications in skeletal muscle tissues are reflected by major alterations in protein expression patterns [35]. VPA has been well known as a potent histone deacetylase (HDAC) inhibitor (HDI) [4], [10], [11]. As mentioned, VPA exerts its teratogenic effect by inhibiting histone deacetylases and by binding to the RA receptor [10]. VPA induced increased Aldh1a2 expression in the somites and decreased expression in the branchialarches [16].

Nuclear acetyltransferases promote and HDIs enhance muscle differentiation [36], while MyoD regulates muscle differentiation [37]. The function of MyoD is to commit mesoderm cells to a skeletal lineage during development and regulates muscle repair [37].

Considerable changes in the fiber type ratio occur as a result of physiological adaptations, in association with many muscular disorders and during the natural aging process [38]. MLC1 is expressed in slow but not fast skeletal muscles [39]. In fast-to-slow muscle transitions during muscle aging, proteomic profiling has clearly established an age-related shift to slower protein isoforms of myosin heavy chain (MHC) and myosin light chain (MLC) [40]. Endurance exercise, chronic low-frequency stimulation, hyper-excitability and aging usually trigger fast-to-slow muscle transformation [41]. Alternatively, slow-to-fast muscle transitions can be typically observed in disuse atrophy, microgravity and extended periods of bed rest [41]. Thus, our results implicated the close association of the hemorrhagic liposis with muscular atrophy which can be elicited by slow-to-fast muscle transitions.

VPA can directly upregulate gene *MLC1* and at the same time indirectly stimulate the active MCL (i.e. the dephosphorylated MCL) [27], [42]. Carnitine palmitoyl transferase (*CPT1*) deficiency has been considered to be one of the most common causes of isolated rhabdomyolysis [27], [42]. Similar result has been found by [9].

### Role of gene ALB (serum albumin precursor)

Decreased serum albumin concentrations following the VPA therapy have been identified in multiple studies [43]. Based on available literature, VPA appears to inhibit an enzyme(s) either directly or indirectly involved with albumin synthesis or albumin gene expression [43]. VPA may indirectly inhibit protein synthesis by interfering with the urea cycle leading to decreased ornithine concentrations, resulting in alterations in albumin synthesis and release [43]

### Role of gene BHMT (betaine-homocysteine S-methyltransferase)

VPA impaired methionine cycle. The remethylation process took place via two independent pathways. One is 5-mTHF-dependent [3], [4], [20], [21] pathway acting as a disrupter of methylene tetrahydrofolate reductase (MTHFR) [18], [19], and the other, the folate-independent (or the betaine-dependent) remethylation pathway if homocysteine is present. The latter proceeds by help of betaine-homocysteine methyltransferase (BHMT) that utilizes a methyl group from betaine to form dimethylglycine and methionine. The alteration of methionine cycle by VPA could be the common mechanism underlying the hepatotoxic, teratogenic and antifolate effects of the drug [25]. Overaccumulated serum SAM [4] downregulates BHMT expression in HepG2 cells in part by inducing NFκB, a repressor for the human BHMT gene [44]. To our believe, we are the first reporting that VPA directly inhibits the folate-independent (or the betaine-dependent) remethylation pathway. The result also mirrored the potential teratogenic nature of VPA.

### Role of gene PKM2 (pyruvate kinase muscle isozyme 2)

Hereditary spherocytosis (HS) and pyruvate kinase (PK) deficiency are the most common causes of congenital hemolytic anemia [45]. Patients with congenital haemolytic anemia was reported to be associated with erythrocyte PK deficiency [46]. Erythrocytic PK deficiency, first documented in Basenjis, is the most common inherited erythroenzymopathy in dogs [47]. Interestingly, although VPA alone did not affect gene *PKM2* at all (Fig. 4E), RV (at 2.0 µM) and Vit E (at 0.2, 2.0 µM), either used alone or cotherapy with VPA, all upregulated *PKM2* (Fig. 4E), underlying the suppression by VPA occurring merely at the proteomic level (spot No. 74 in Fig. 2, Fig. 3).

It is worthy noting, protein-tyrosine phosphatase 1B (PTP1B) acts as a negative regulator of the insulin signaling pathway [48]–[50]. The finding that PKM2 acts as a novel substrate of PTP1B also provides new insights into the regulation of adipose PKM2 activity [51].

Alternatively, VPA evoked homocysteine accumulation through impairment of methionine cycle [4]. Homocysteine decreased the viability of mitochondrion and the activities of PKM2 and creatine kinase [52]. Hyperhomocysteinemia severely affected patients with several manifestations including a variable degree of motor dysfunction [52]. Likewise, VPA induced severe muscular atrophy with hemorrhagic liposis in pecking muscles (cervical muscles) [4], [8].

### Role of gene FLNC (filamin C)

Deficiency of protein FLNC has been associated with muscle weakness [35]. Pathophysiology of most metabolic myopathies is related to the impairment of energy production or to abnormal production of reactive oxygen species (ROS) [27], [42].

Literature indicates that skeletal muscle-specific deletion of both HDAC1 and HDAC2 results in perinatal lethality of a subset of mice, accompanied by mitochondrial abnormalities and sarcomere degeneration [53]. VPA is a potent HDAC inhibitor, while HDAC 1 and 2 control skeletal muscle homeostasis and autophagy flux in mice [53]. VPA would definitely play a part of role in the generation of myopathy. HDAC1 and HDAC2 play the role in maintenance of skeletal muscle structure and function and some pathological conditions [53].

Metabolic myopathies frequently can lead to extra-neuromuscular disorders [42]. Embryos at earlier stage are more susceptible to VPA therapy [9], [54]. Apparently, the change of proteomic profiling in reality is not only limited to age, rather is associated with individual innate “physiological condition” like nutritional status, stresses, diseases, medication and exercise [9], [54].

Finally, the problem about the role of HDAC has attracted our attention. Could RV and vit E play the same role as VPA? Astonishingly, RV acts as a pan-HDAC inhibitor alters the acetylation status of histone [corrected] proteins in human-derived hepatoblastoma cells. In vivo chicken embryotoxicity assays demonstrated severe toxicity of RV at high concentrations [55].

Other dietary agents such as metabolites of vitE have structural features compatible with HDAC inhibition [56]. The ability of dietary compounds to de-repress epigenetically silenced genes in cancer cells, and to activate these genes in normal cells, has important implications for cancer prevention and therapy. In a broader context, there is growing interest in dietary HDAC inhibitors and their impact on epigenetic mechanisms affecting other chronic conditions, such as cardiovascular disease, neurodegeneration and aging [56].

### Materials

Bovine serum albumin (BSA), sodium dodecyl sulfate (SDS), Coomassie Brilliant Blue R (CBR) and N,N,N′,N′-tetramethyl-ethylenediamine (TEMED) were purchased from Sigma Co. (St. Louis, MO, USA). Bio-Rad protein assay kits, Bio-Rad protein standards solutions, amphoteric solutions (Bio-Lyte 3–10 Ampholyte), 30% bisacrylamide, 19∶1, and mineral oil were products of Bio-Rad (Hercules, California, USA). The 2-D Clean-up Kit was a product of GE Healthcare Bio-Science Corp. (Piscataway, USA). Anti-rabbit IgG HRP-linked antibodies, Tris buffered saline with Tween-20 (TBST-10X) and cell lysis buffer were provided by Cell Signaling Technology Co. (Massachusetts, USA). ProteoJETTM Mammalian Cell Lysis Reagent, ProteoBlockTM and Protease Inhibitor Cocktail were products of Fermentas Co. (USA). Trypsin was a product of Promega (Madison, WI, USA). Acetonitrile (ACN) was supplied by JT Baker Chemical Products Trading (Shanghai) Co., Ltd. (China). Other chemicals not denoted were provided by Sigma Co. (St. Louis, MO, USA). All reagents used in this experimentation were prepared as previously described [5].

### Limitations of the study

Due the limitation of feasible kits source and its related suppliers related to the chicken embryo model, our research work has been definitely so difficult and limited. In addition, the expressions of genes and signal proteins are damping sequentially and rapidly in dose- and age-dependent manner (as can be expected), we would have missed a lot of valuable informations, we guess.

Thus, in addition to the downregulation of *CPT1* and the upregulation of *ACC* as previously reported [8], we conclude that VPA tends to downregulate *PEBP1* and *BHMT* (*p*<0.05) and upregulate *MYL1, ALB and FLNC* (*p*<0.05) at early embryonic stage without affecting *PKM2*, implicating the direct inhibition on the folate-independent remethylation pathway. These genes are closely related with metabolic myopathies, myogenesis, albumin gene expression, and haemolytic anemia. In addition, VPA directly inhibits the folate-independent (or the betaine-dependent) remethylation pathway, underlying the action mechanism of VPA to induce hemorrhagic myoliposis. RV and vit E are effective for alleviation of such adverse effects.

## Supporting Information

S1 Table LC/MS/MS identification of proteins expressed in chicken cervical muscle samples. Data were confirmed by at least six determinations (for review only).
(DOCX)
