# Supplementary material for: The Proteomic and Genomic Teratogenicity Elicited by Valproic Acid Is Preventable with Resveratrol and α-Tocopherol
Source: PLoS One. 2014 Dec 31;9(12):e116534. doi: 10.1371/journal.pone.0116534 (PMC4281235; doi:10.1371/journal.pone.0116534)
Supplement: S1 Table — LC/MS/MS identification of proteins expressed in chicken cervical muscle samples. Data were confirmed by at least six determinations (for review only). (DOCX) [file pone.0116534.s001.docx]

**Table S1. LC/MS/MS identification of proteins expressed in chicken cervical muscle samples.** Data were confirmed by at least six determinations.

| **Spot No.** |  | **Accession No.** | | | | | **Protein Description** | | **MOWSE score** | | | **Experimental Mr/pl** | | | **Theoretical Mr/pl** | | **Protein**  **expression in** | |
| --- | --- | --- | --- | --- | --- | --- | --- | --- | --- | --- | --- | --- | --- | --- | --- | --- | --- | --- |
| 1 | 1 | gi∣310772215 | | | | | phosphatidylethanolamine-binding protein 1[Gallus gallus] | | 396 | | | n.a./n.a. | | | 21.12/6.96 | |  | |
|  | 2 | gi∣126608 | | | | | RecName:Full=Lysozyme C;AltName:Full=1.4-beta-N-acetylmuramidase C;AltName:Full=Allergen Gal d Ⅳ;AltName:Allergen=Gal d 4;Flags:Precursor | | 164 | | |  | | | 16.74/9.37 | |  | |
|  | 3 | gi∣422920046 | | | | | Chain l,Structure Of The Anti-ptau Fab (pt231/ps235_1)  In Complex With Phosphoepitope Pt231/ps235 | | 117 | | |  | | | 22.43/5.13 | |  | |
|  | 4 | gi∣58332100 | | | | | protease.serine.2 (trypsin 2) precursor [Xenopus (Silurana)tropicalis] | | 115 | | |  | | | 26.13/6.38 | |  | |
|  | 5 | gi∣63052 | | | | | unnamed protein product [Gallus gallus] | | 90 | | |  | | | 17.69/5.35 | |  | |
|  | 6 | gi∣449272815 | | | | | Alpha-2-macroglobulin-like protein 1,partial [Columbalivia] | | 71 | | |  | | | 159.69/6.39 | |  | |
|  | 7 | gi∣63018 | | | | | beta-actin [Gallus gallus] | | 57 | | |  | | | 42.08/5.29 | |  | |
|  | 8 | gi∣465958060 | | | | | Kallikrein-11 [Chelonia mydas] | | 57 | | |  | | | 27.28/7.49 | |  | |
|  | 9 | gi∣363740904 | | | | | PREDICTED:sodium-independent sulfate anion transporter [Gallus gallus] | | 53 | | |  | | | 65.25/8.62 | |  | |
|  | 10 | gi∣422920045 | | | | | Chain H,Structure Of The Anti-ptau Fab (pt231/ps235_1) In Complex With Phosphoepitope Pt231/ps235 | | 49 | | |  | | | 23.92/8.52 | |  | |
| 3 | 1 | gi∣129293 | | | | | RecName:Full=Ovalbumin;AltName:Full=Allergen Gal d Ⅱ;AltName:Full=Egg albumin;AltName Full:Plakalbumin;AltName:Allergen=Gal d 2 | | 334 | | | n.a./n.a. | | | 43.20/5.19 | |  | |
|  | 2 | gi∣326915675 | | | | | PREDICTED:superoxide dismutase [Mn],mitochondrial-like [Meleagins gallopavo] | | 159 | | |  | | | 26.34/8.58 | |  | |
|  | 3 | gi∣422920046 | | | | | Chain l,Structure Of The Anti-ptau Fab (pt231/ps235_1) In Complex With Phosphoepitope Pt231/ps235 | | 107 | | |  | | | 22.43/5.13 | |  | |
|  | 4 | gi∣58332100 | | | | | protease.serine.2 (trypsin 2) precursor [Xenopus (Silurana) tropicalis] | | 93 | | |  | | | 26.13/6.38 | |  | |
|  | 5 | gi∣126608 | | | | | RecName:Full=Lysozyme C;AltName:Full=1,4-beta-N-acetylmuramidase C;AltName Full=Allergen Gal d Ⅳ;AltName:Allergen=Gal d 4;Flags:Precursor | | 82 | | |  | | | 16.74/9.37 | |  | |
|  | 6 | gi∣449272815 | | | | | Alpha-2-macroglobulin-like protein 1 , partial [Columbalivia] | | 71 | | |  | | | 159.69/6.39 | |  | |
|  | 7 | gi∣465958060 | | | | | Kallikrein-11 [Chelonia mydas] | | 52 | | |  | | | 27.28/7.49 | |  | |
|  | 8 | gi∣422920045 | | | | | Chain H,Structure Of The Anti-ptau Fab (pt231/ps235_1) In Complex With Phosphoepitope | | 50 | | |  | | | 23.92/8.52 | |  | |
|  |  |  | | | | | Pt231/ps235 | |  | | |  | | |  | |  | |
|  | 9 | gi∣327277730 | | | | | PREDICTED:desmoplakin-like [Anolis carolinensis] | | 48 | | |  | | | 333.02/6.38 | |  | |
|  | 10 | gi∣301627197 | | | | | PREDICTED:LOW QUALITY PROTEIN:SWI/SNF complex subunit SMARCC2 [Xenopus (Silurana) tropicalis] | | 47 | | |  | | | 124.56/5.43 | |  | |
| 9 | 1.1 | gi∣55584149 | | | | | RecName:Full=Myosin light chain 1, skeletal muscle isoform;AltName:Full=Alkali myosin light chain 1;Short=MLC-1;AltName:Full=Myosin light chain 1f;AltName:Full=Myosin light chain alkali 1;Short=Myosin light chain A1;AltName:Full=Skeletal | | 965 | | | n.a./n.a. | | | 20.94/4.96 | |  | |
|  | 1.2 | gi∣1772516 | | | | | cardiac myosin alkali light chain [Gallus gallus] | | 274 | | |  | | | 16.23/5.13 | |  | |
|  | 2 | gi∣129293 | | | | | RecName:Full=Ovalbumin;AltName:Full=Allergen Gal d Ⅱ;AltName:Full=Egg albumin;AltName:Full=Plakalbumin;AltName:Allergen=Gal d 2 | | 243 | | |  | | | 43.20/5.19 | |  | |
|  | 3 | gi∣211146 | | | | | apolipoprotein A-1 precursor [Gallus gallus] | | 221 | | |  | | | 30.67/5.97 | |  | |
|  | 4 | gi∣157878404 | | | | | Chain A,Hydrogen And Hydration Of Hen Egg-White Lysozyme Determined By Neutron Diffraction | | 172 | | |  | | | 14.74/9.20 | |  | |
|  | 5 | gi∣58332100 | | | | | protease, serine, 2 (trypsin 2) precursor [Xenopus (Silurana) tropicalisd] | | 107 | | |  | | | 26.13/6.38 | |  | |
|  | 6 | gi∣4492722815 | | | | | Alpha-2-macroglobulin-like protein 1 , partial [Columbalivia] | | 80 | | |  | | | 159.69/6.39 | |  | |
|  | 7 | gi∣422920046 | | | | | Chain l, Structure Of The Anti-ptau Fab (pt231/ps235_1) In Complex With Phosphoepitope Pt231/ps235 | | 72 | | |  | | | 22.43/5.13 | |  | |
|  | 8 | gi∣465958060 | | | | | Kallilrein-11 [Chelonia mydas] | | 59 | | |  | | | 27.28/7.49 | |  | |
|  | 9 | gi∣46195455 | | | | | cathepsin B precursor [Gallus gallus] | | 59 | | |  | | | 38.48/5.74 | |  | |
|  | 10 | gi∣422920045 | | | | | Chain H, Structure Of The Anti-ptau Fab (pt231/ps235_1) In Complex With Phosphoepitope Pt231/ps235 | | 48 | | |  | | | 23.92/8.52 | |  | |
| 10 | 1.1 | gi∣326931250 | | | | | PREDICTED:heat shock protein beta-1-like [Meleagris gallopavo] | | 452 | | | n.a./n.a. | | | 21.89/6.23 | |  | |
|  | 1.2 | gi∣45384222 | | | | | heat shock protein beta-1 [Gallus gallus] | | 434 | | |  | | | 21.72/5.77 | |  | |
|  | 2 | gi∣34221747 | | | | | triosephosphate isomerase [Meleagris gallopavo] | | 134 | | |  | | | 22.75/6.19 | |  | |
|  | 3.1 | gi∣113271 | | | | | RecName:Full=Actin, cytoplasmic;AltName:Full=Beta actin | | 119 | | |  | | | 42.16/5.29 | |  | |
|  | 3.2 | gi∣326920308 | | | | | PREDICTED:actin, alpha skeletal muscle B-like [Meleagris gallopavo] | | 89 | | |  | | | 42.34/5.16 | |  | |
|  | 4 | gi∣211146 | | | | | apolipoprotein A-1 precursor [Gallus gallus] | | 118 | | |  | | | 30.67/5.97 | |  | |
|  | 5 | gi∣58332100 | | | | | protease, serine, 2 (trypsin 2) precursor [Xenopus (Silurana) tropicalis] | | 97 | | |  | | | 26.13/6.38 | |  | |
|  | 6 | gi∣422920046 | | | | | Chain l, Structure Of The Anti-ptau Fab (pt231/ps235_1) In Complex With Phosphoepitope Pt231/ps235 | | 83 | | |  | | | 22.43/5.13 | |  | |
|  | 7 | gi∣45361639 | | | | | methionine adenosyltransferase Ⅱ, alpha [Xenopus (Silurana) tropicalis] | | 61 | | |  | | | 43.98/6.02 | |  | |
|  | 8 | gi∣465957486 | | | | | Tubulin alpha-1B chain [Chelonia mydas] | | 53 | | |  | | | 171.85/5.56 | |  | |
|  | 9 | gi∣175378485 | | | | | ribosomal protein L4 [Xenopus muellen] | | 43 | | |  | | | 25.61/11.57 | |  | |
|  | 10 | gi∣326919110 | | | | | PREDICTED:plasma kallikrein-like [Meleagris gallopavo] | | 41 | | |  | | | 83.73/7.40 | |  | |
| 13 | 1.1 | gi∣230359 | | | | | Chain A, Structure Of Triose Phosphate Isomerase From Chicken Muscle | | 837 | | | n.a./n.a. | | | 26.76/7.26 | |  | |
|  | 1.2 | gi∣34221912 | | | | | trisephosphste isomerase [Phasianus colchicus] | | 814 | | |  | | | 22.77/6.19 | |  | |
|  | 1.3 | gi∣326912719 | | | | | PREDICTED:triosephosphate isomerase-like [Meleagris gallopavo] | | 770 | | |  | | | 24.29/6.45 | |  | |
|  | 1.4 | gi∣136061 | | | | | RecName:Full=Triosephosphate isomerase;Short=TIM;AitName:Full=Triose-phosphate isomerase | | 351 | | |  | | | 27.00/5.39 | |  | |
|  | 2 | gi∣129293 | | | | | RecName:Full=Ovalbumin;AltName:Full=Allergen Gal d Ⅱ;AltName:Full=Egg albumin;AltName:Full=Plakalbumin;AltName:Allergen=Gal d 2 | | 360 | | |  | | | 43.20/5.19 | |  | |
|  | 3 | gi∣326933524 | | | | | PREDICTED:selenium-binding protein 1-like [Meleagris gallopavo] | | 153 | | |  | | | 71.89/6.53 | |  | |
|  | 4 | gi∣126608 | | | | | RecName:Full=Lysozyme C;AltName:Full=1,4-beta-N-acetylmuramidase C;AltName:Full=Allergen Gal d Ⅳ;AltName:Allergen=Gal d 4;Flags: Precursor | | 139 | | |  | | | 16.74/9.37 | |  | |
|  | 5 | gi∣58332100 | | | | | protease, serine, 2 (trypsin 2) precursor [Xenopus (Silurana) tropicalis] | | 96 | | |  | | | 26.13/6.38 | |  | |
|  | 6 | gi∣326933528 | | | | | PREDICTED:selenium-binding protein 1-like [Meleagris gallopavo] | | 73 | | |  | | | 48.62/6.88 | |  | |
|  | 7 | gi∣422920046 | | | | | Chain l, Structure Of The Anti-ptau Fab (pt231/ps235_1) In Complex With Phosphoepitope Pt231/ps235 | | 72 | | |  | | | 22.43/5.13 | |  | |
|  | 8 | gi∣ 478431053 | | | | | 3,2-trans-enoyl-CoA isomerase, mitochondrial [Gallus gallus] | | 70 | | |  | | | 34.56/9.30 | |  | |
|  | 9 | gi∣89891990 | | | | | ubiquitin C Ⅱ[Anser anser] | | 69 | | |  | | | 30.39/6.38 | |  | |
|  | 10 | gi∣422920045 | | | | | Chain H, Structure Of The Anti-ptau Fab (pt231/pa235_1) In Complex With Phosphoepitope Pt231/ps235 | | 57 | | |  | | | 23.92/8.52 | |  | |
| 42 | 1.1 | gi∣330417943 | | | | | fructose-bisphosphate aldolase C[Gallus gallus] | | 1555 | | | n.a./n.a. | | | 39.74/6.20 | |  | |
|  | 1.2 | gi∣ 409191 | | | | | aldolase A, partial [Gallus gallus] | | 65 | | |  | | | 4.44/6.95 | |  | |
|  | 2 | gi∣57525158 | | | | | septin-2 [Gallus gallus] | | 170 | | |  | | | 40.37/6.12 | |  | |
|  | 3 | gi∣422920046 | | | | | Chain l,Structure Of The Anti-ptau Fab (pt231/ps235_1) In Complex With Phosphoepitope Pt231/ps235 | | 129 | | |  | | | 22.43/5.13 | |  | |
|  | 4 | gi∣449270735 | | | | | Actin, aortic, smooth muscle [Columba livia] | | 113 | | |  | | | 42.37/5.17 | |  | |
|  | 5 | gi∣58332100 | | | | | protease, serine, 2 (trypsin 2) precursor [Xenopus (Silurana) tropicalis] | | 103 | | |  | | | 26.13/6.38 | |  | |
|  | 6 | gi∣327286458 | | | PREDICTED: s-adenosylmethionine synthase isoform type-2-like [Anolis carolinensis] | | | | | 94 | | |  | | | 43.61/6.92 | |  |
|  | 7 | gi∣45382875 | | | Creatine kinase M-type [Gallus gallus] | | | | | 81 | | |  | | | 43.53/6.50 | |  |
|  | 8 | gi∣34811330 | | | Chain A, Crystal Structure Of S-ovalbumin At 1.9 Angstrom Resolution | | | | | 77 | | |  | | | 43.18/5.20 | |  |
|  | 9 | gi∣50732421 | | | PREDICVED: phosphotriesterase-related protein [Gallus gallus] | | | | | 72 | | |  | | | 39.43/6.10 | |  |
|  | 10 | gi∣449272815 | | | Alpha-2-macroglobulin-like protein 1, partial [Columba livia] | | | | | 66 | | |  | | | 159.69/6.39 | |  |
| 43 | 1.1 | gi∣330417943 | | | Fructose-bisphosphate aldolase C [Gallus gallus] | | | | | 1166 | | | n.a./n.a. | | | 39.74/6.20 | |  |
|  | 1.2 | gi∣327280689 | | | PREDICTED: fructose-bisphosphate aldolase A-like [Anolis carolinensis] | | | | | 91 | | |  | | | 39.96/8.67 | |  |
|  | 1.3 | gi∣409191 | | | Aldolase A, partial [Gallus gallus] | | | | | 61 | | |  | | | 4.44/6.95 | |  |
|  | 2 | gi∣363733121 | | | PREDICTED: peptidyl-prolyl cis-trans isomerase D [Gallus gallus] | | | | | 119 | | |  | | | 40.85/5.98 | |  |
|  | 3 | gi∣58332100 | | | Protease, serine, 2(trypsin 2) precursor [Xenopus (Silurana) tropicalis] | | | | | 113 | | |  | | | 26.13/6.38 | |  |
|  | 4 | gi∣422920046 | | | Chain I, Structure Of The Anti-ptau Fab (pt231/ps235_1) In Complex With Phosphoepitope Pt231/ps235 | | | | | 92 | | |  | | | 22.43/5.13 | |  |
|  | 5 | gi∣63052 | | | Unnmed protein product [Gallus gallus] | | | | | 91 | | |  | | | 17.69/5.35 | |  |
|  | 6 | gi∣345100466 | | | Chain A, Hen Egg White Lysozyme With A Isoaspartate Residue | | | | | 88 | | |  | | | 14.76/9.46 | |  |
|  | 7 | gi∣45382875 | | | Creatine kinase M-type [Gallus gallus] | | | | | 74 | | |  | | | 43.53/6.50 | |  |
|  | 8 | gi∣326935589 | | | PREDICTED:macrophage-capping protein-like, partial [Meleagris gallopavo] | | | | | 70 | | |  | | | 27.45/5.85 | |  |
|  | 9 | gi∣71896529 | | | Eukaryotic translation initiation factor 3 subunit H [Gallus gallus] | | | | | 66 | | |  | | | 39.68/6.03 | |  |
|  | 10 | gi∣449272815 | | | Alpha-2-macroglobulin-like protein 1, partial [Columba livia] | | | | | 63 | | |  | | | 159.69/6.39 | |  |
| 55 | 1 | gi∣50755288 | | | PREDICTED: betaine—homocysteine S-methyltransferase 1 [Gallus gallus] | | | | | 1764 | | | n.a./n.a. | | | 45.55/7.56 | |  |
|  | 2.1 | gi∣45384486 | | | Phosphoglycerte kinase [Gallus gallus] | | | | | 183 | | |  | | | 45.09/8.31 | |  |
|  | 2.2 | gi∣483518353 | | | Phosphoglycerate kinase, partial [Anas platyrhynchos] | | | | | 181 | | |  | | | 39.52/6.79 | |  |
|  | 3 | gi∣57529492 | | | 3-ketoacyl-CoA thiolase, mitochondrial [Gallus gallus] | | | | | 162 | | |  | | | 42.17/8.02 | |  |
|  | 4 | gi∣363745828 | | | PREDICTED: glutaryl-CoA dehydrogenase, mitochondrial-like, partial [Gallus gallus] | | | | | 147 | | |  | | | 25.70/6.67 | |  |
|  | 5 | gi∣326933955 | | | PREDICTED: 26S protease regulatory subunit 8-like [Meleagris gallopavo] | | | | | 139 | | |  | | | 46.31/7.11 | |  |
|  | 6 | gi∣422920046 | | | Chain I, Structure Of The Anti-ptau Fab (pt231/ps235_1) In Complex With Phosphoepitope Pt231/ps235 | | | | | 135 | | |  | | | 22.43/5.13 | |  |
|  | 7 | | gi∣58332100 | | | | Protease, serine,2(trypsin 2) precursor [Xenopus (Silurana) tropicalis] | 117 | | | |  | | | 26.13/6.38 | |  | |
|  | 8.1 | | gi∣297296096 | | | | PREDICTED: actin. Alpha cardiac muscle 1 [Macaca mulatta] | 97 | | | |  | | | 37.54/5.27 | |  | |
|  | 8.2 | | gi∣113271 | | | | RecName: Full=Actin, cytoplasmic 1;AltName: Full=Beta actin | 95 | | | |  | | | 42.16/5.29 | |  | |
|  | 9 | | gi∣63052 | | | | Unnamed protein product [Gallus gallus] | 84 | | | |  | | | 17.69/5.35 | |  | |
|  | 10 | | gi∣118093509 | | | | PREICTED: isocitrate dehydrogenase [NADP] cytoplasmic [Gallus gallus] | 79 | | | |  | | | 46.97/8.02 | |  | |
| 65 | 1 | | gi∣46048765 | | | | Beta-enolase [Gallus gallus] | 705 | | | | n.a./n.a. | | | 47.57/7.28 | |  | |
|  | 2 | | gi∣50755288 | | | | PREDICTED: betaine—homocysteine S-methyltransferase 1 [Gallus gallus] | 139 | | | |  | | | 45.55/7.56 | |  | |
|  | 3 | | gi∣422920046 | | | | Chain I, Strusture Of The Anti-ptau Fab (pt231/ps235_1) In Complex With Phosphoepitope Pt231/ps235 | 109 | | | |  | | | 22.43/5.13 | |  | |
|  | 4 | | gi∣58332100 | | | | protease, serine, 2(trypsin 2) precursor [Xenopus (Silurana) tropicalis] | 100 | | | |  | | | 26.13/6.38 | |  | |
|  | 5 | | gi∣449488145 | | | | PREDICTED:LOW QUALITY PROTEIN: actin, cytoplasmic type 5 [Taeniopygia guttata] | 94 | | | |  | | | 34.98/4.96 | |  | |
|  | 6 | | gi∣45361639 | | | | Methionine adenosyltransferase Ⅱ, alpha [Xenopus (Silurana) tropicalis] | 95 | | | |  | | | 43.98/6.02 | |  | |
|  | 7 | | gi∣126608 | | | | RecName: Full=Lysozyme C; AltName: Full=1,4-beta-N-acetylmuramidase C; AltName: Full=Allergen Gal d Ⅳ; AltName: Allergen=Gal d 4; Falgs: Precursor | 73 | | | |  | | | 16.74/9.37 | |  | |
|  | 8 | | gi∣465958060 | | | | Kallikrein-11 [Chelonia mydas] | 50 | | | |  | | | 27.28/7.49 | |  | |
|  | 9 | | gi∣422920045 | | | | Chain H, Structure Of The Anti-ptau Fb (pt231/ps235_1) In Complex With Phosphoepitope Pt231/ps235 | 47 | | | |  | | | 23.92/8.52 | |  | |
|  | 10 | | gi∣449272815 | | | | Alpha-2-macroglobulin-like protein 1, partial [Columba livia] | 41 | | | |  | | | 159.69/6.39 | |  | |
| 66 | 1.1 | | gi∣46048765 | | | | Beta-enolase [Gallus gallus] | 475 | | | | n.a./n.a. | | | 47.57/7.28 | |  | |
|  | 1.2 | | gi∣326932384 | | | | PREDICTED: alpha-enolase-like [Meleagris gallopavo] | 404 | | | |  | | | 47.65/6.30 | |  | |
|  | 2 | | gi∣28566340 | | | | Ovalbumin [Gallus gallus] | 228 | | | |  | | | 43.22/5.19 | |  | |
|  | 3 | | gi∣45384504 | | | | Sarcalumenin precursor [Gallus gallus] | 183 | | | |  | | | 54.57/6.59 | |  | |
|  | 4 | | gi∣1351295 | | | | RecName: Full=Ovotransferrin; AltName: Full- Allergen Gal d Ⅲ; AltName: Full=Conalbumin; AltName: Full=Serum transferrin; AltName: Allergen=Gal d 3; Flags: Precursor | 133 | | | |  | | | 79.55/6.85 | |  | |
|  | 5 | | gi∣58332100 | | | | Protease, serine, 2(trypsin 2) precursor [enopus (Silurana) tropicalis] | 109 | | | |  | | | 26.13/6.38 | |  | |
|  | 6 | | | gi∣422920046 | | | Chain I, Structure Of The Anti-ptau Fab (pt231/ps235_1) In Complex With Phosphoepitope Pt231/ps235 | | 99 | | |  | | | 22.43/5.13 | |  | |
|  | 7 | | | gi∣61098378 | | | Aspartyl aminopeptidase [Gallus gallus] | | 92 | | |  | | | 52.57/6.55 | |  | |
|  | 8 | | | gi∣732995 | | | Pre-fibrinogen alpha subunit [Gallus gallus] | | 72 | | |  | | | 56.77/6.82 | |  | |
|  | 9 | | | gi∣126608 | | | Rec Name: Full=Lysozym C; AltName: Full=1,4-beta-N-acetylmuramidase C; AltName: Full=Allergen Gal d Ⅳ; AltName: Allergen=Gal d 4; Flags: Precursor | | 66 | | |  | | | 16.74/9.37 | |  | |
|  | 10 | | | gi∣50754375 | | | PREDICTED: cytochrome b-c1 complex subunit 1, mitochondrisl [Gallus gallus] | | 55 | | |  | | | 53.41/6.58 | |  | |
| 67 | 1 | | | gi∣45383033 | | | Filamin-C [Gallus gallus] | | 465 | | | n.a./n.a. | | | 283.23/5.93 | |  | |
|  | 2 | | | gi∣119338 | | | RecName: Full=Alpha-enolase; AltName: Full=2-phospho-D-glycerate hydro-lyase; AltName: Full=Tau-crystallin | | 145 | | |  | | | 47.61/6.37 | |  | |
|  | 3 | | | gi∣58332100 | | | Protease, serine, 2(trypsin 2) precursor [Xenopus (Silurana) tropicalis] | | 101 | | |  | | | 26.13/6.38 | |  | |
|  | 4 | | | gi∣422920046 | | | Chain I, Structure Of The Anti-ptau Fab (pt231/ps235_1) In Complex With Phosphoepitope Pt231/ps235 | | 83 | | |  | | | 22.43/5.13 | |  | |
|  | 5 | | | gi∣63052 | | | Unnamed protein product [Gallus gallus] | | 79 | | |  | | | 7.69/5.35 | |  | |
|  | 6 | | | gi∣50755288 | | | PREDICTED: betaine—homocysteine S-methyltansferase 1 [Gallus gallus] | | 65 | | |  | | | 45.55/7.56 | |  | |
|  | 7 | | | gi∣465958060 | | | Kallikrein-11 [Chelonia mydas] | | 55 | | |  | | | 27.28/7.49 | |  | |
|  | 8 | | | gi∣63018 | | | Beta0actin [Gallus gallus] | | 57 | | |  | | | 42.08/5.29 | |  | |
|  | 9 | | | gi∣89274064 | | | Haptoglobin-like protein [Phalacrocorax carbo] | | 53 | | |  | | | 37.66/5.34 | |  | |
|  | 10 | | | gi∣61098378 | | | Aspartyl aminopeptidase [Gallus gallus] | | 49 | | |  | | | 52.57/6.55 | |  | |
| 70 | 1 | | | gi∣71897021 | | | Dihydrolipoyl dehydrogenase, mitochondrial [Gallus gallus] | | 130 | | | n.a./n.a. | | | 54.57/8.19 | |  | |
|  | 2 | | | gi∣422920046 | | | Chain I, Structure Of The Anti-ptau Fab (pt231/ps235_1) In Complex With Phosphoepitope Pt231/ps235 | | 113 | | |  | | | 22.43/5.13 | |  | |
|  | 3 | | | gi∣465982130 | | | Kinesin-like protein KIF23, partial [Chelonia mydas] | | 5 | | |  | | | 102.66/7.84 | |  | |
|  | 4 | | | gi∣327259855 | | | PREDICTED: doublecortin domain-containing protein 5-like [Anoli carolinensis] | | 41 | | |  | | | 107.09/7.73 | |  | |
|  | 5 | | | gi∣58331950 | | | Cytosol aminopeptidase [Xenopus (Silurana) tropicalis] | | 37 | | |  | | | 56.75/8.45 | |  | |
|  | 6 | | | gi∣362740904 | | | PREDICTED: sodium-independent sulfate anion transporter [Gallus gallus] | | 37 | | |  | | | 65.25/8.62 | |  | |
| 74 | 1.1 | | | gi∣45382651 | | | Pyruvate kinase muscle isozyme [Gallus gallus] | | 2934 | | | n.a./n.a. | | | 58.43/7.29 | |  | |
|  | 1.2 | | | gi∣465992345 | | | Pyruvate kinse muscle isozyme [Chelonis mydas] | | 1443 | | |  | | | 67.71/6.82 | |  | |
|  | 2 | | | gi∣129293 | | | RecName: Full=Ovalbumin; AltName: Full=Allergen Gal d Ⅱ; AltName: Full=Egg albumin; AltName: Full=Plakalbumin; AltName: Allergen=Gal d 2 | | 188 | | |  | | | 43.20/5.19 | |  | |
|  | 3 | | gi∣1351295 | | | RecName: Full=Ovotransferrin; AltName: Full=Allergen Gal d Ⅲ; AltName: Full=Conalbumin; AltName: Full=Serum transferrin; AltName: Allergen=Gal d 3; Flags: Precursor | | 112 | | |  | | | 79.55/6.85 | | | |  |
|  | 4 | | gi∣345100466 | | | Chain A, Hen Egg White Lysozyme With A Isoaspartate Residue | | 109 | | |  | | | 14.76/9.46 | | | |  |
|  | 5 | | gi∣50750688 | | | PREDICTED: protein disulfide-isomerase A5 [Gallus gallus] | | 104 | | |  | | | 61.32/8.24 | | | |  |
|  | 6 | | gi∣58332100 | | | Protease, serine, 2(trypsin 2) precursor [Xenopus (Silurana) tropicalis] | | 103 | | |  | | | 26.13/6.38 | | | |  |
|  | 7 | | gi∣422920046 | | | Chain I, Structure Of The Anti-ptau Fab (pt231/ps235_1) In Complex With Phosphoepitope Pt231/ps235 | | 92 | | |  | | | 22.43/5.23 | | | |  |
|  | 8 | | gi∣60302808 | | | UDP-glucose 6-dehydrogenase [Gallus gallus] | | 90 | | |  | | | 55.77/6.99 | | | |  |
|  | 9 | | gi∣449272815 | | | Alpha-2-macroglobulin-like protein 1, partial [Colimba livia] | | 79 | | |  | | | 159.69/6.39 | | | |  |
|  | 10 | | gi∣223033 | | | Collagen alpha1 CB7 | | 73 | | |  | | | 24.39/9.39 | | | |  |
| 88 | 1 | | gi∣45383974 | | | Serum albumin precursor [Gallus gallus] | | 937 | | | n.a./n.a. | | | 71.87/5.51 | | | |  |
|  | 2 | | gi∣326928269 | | | PREDICTED: stress-70 protein, mitochondrial-like [Mleagris gallopavo] | | 444 | | |  | | | 79.55/7.13 | | | |  |
|  | 3.1 | | gi∣30962014 | | | Heat shock protein 70 [Gallus gallus] | | 428 | | |  | | | 70.0/5.66 | | | |  |
|  | 3.2 | | gi∣161408079 | | | Heat shock protein 70B [Alligator mississippiensis] | | 149 | | |  | | | 7.03/5.37 | | | |  |
|  | 4 | | gi∣45382651 | | | Pyruvate kinase muscle isozyme [Gallus gallus] | | 272 | | |  | | | 58.43/7.29 | | | |  |
|  | 5 | | gi∣422920046 | | | Chain I, Structure Of The Anti-ptau Fab (pt231/ps235_1) In Complex With Phosphoepitope Pt231/ps235) | | 158 | | |  | | | 22.43/5.13 | | | |  |
|  | 6 | | gi∣58332100 | | | Protease, serine, 2(trypsin 2) precursor [Xenopus (Silurana) tropicalis] | | 106 | | |  | | | 26.13/6.38 | | | |  |
|  | 7 | | gi∣465958060 | | | Kallikrein-11 [Chelonia mydas] | | 72 | | |  | | | 27.28/7.49 | | | |  |
|  | 8 | | gi∣89274064 | | | Haptoglobin-like protein [Phalacrocorax carbo] | | 65 | | |  | | | 37.66/5.34 | | | |  |
|  | 9 | | gi∣50892955 | | | Iron binding protein [Trachemys scripta elegans] | | 59 | | |  | | | 78.63/7.15 | | | |  |
|  | 10 | | gi∣211907087 | | | Smarcc1, partial [Bombina orientalis] | | 48 | | |  | | | 26.91/4.67 | | | |  |
| 95 | 1.1 | | gi∣83754919 | | | Chain A, Crystal Structure Of Aluminum-Bound Ovotransferrin At 2.15 Angstrom Resolution | | 1831 | | | n.a./n.a. | | | 77.52/6.70 | | | |  |
|  | 1.2 | | gi∣71274079 | | | Ovotransferrin BC TYPE [Gallus gallus] | | 1818 | | |  | | | 79.59/7.08 | | | |  |
|  | 2 | | gi∣63524 | | | Unnamed protein product [Gallus gallus] | | 220 | | |  | | | 54.46/6.84 | | | |  |
|  | 3 | | gi∣118084650 | | | PREDICTED: propionyl-CoA carboxylase alpha chain, mitochondrial [Gallus gallus] | | 152 | | |  | | | 79.25/7.85 | | | |  |
|  | 4.1 | | gi∣161513267 | | | Immunoglobulin heavy chain variable region [Gallus gallus] | | 134 | | |  | | | 13.39/7.83 | | | |  |
|  | 4.2 | | gi∣89000553 | | | Immunoglobulin heavy chain variable region [Gallus gallus] | | 53 | | |  | | | 15.52/4.90 | | | |  |
|  | 5 | gi∣363733434 | | | PREDICTED: alpha-fetoprotein [Gallus gallus] | | | 123 | | |  | | | 71.61/6.94 | | | |  |
|  | 6 | gi∣58332100 | | | Protease, srine 2(trypsin 2) precursor [Xenopus (Silurana) tropicalis] | | | 106 | | |  | | | 26.13/6.38 | | | |  |
|  | 7 | gi∣422920046 | | | Chain I, Stucture Of The Anti-ptau Fab (pt231/ps235_1) In Complex With Phospjoepitope Pt231/ps235 | | | 89 | | |  | | | 2.43/5.13 | | | |  |
|  | 8 | gi∣449272815 | | | Alha-2-macroglobulin-like protein 1, partial [Columba livia] | | | 79 | | |  | | | 159.69/6.39 | | | |  |
|  | 9 | gi∣118093904 | | | PREDICTED: glycerol-3-phosphate dehydrogenase mitochondrial [Gallus gallus] | | | 76 | | |  | | | 81.16/7.58 | | | |  |
|  | 10 | gi∣57529561 | | | GMP synthase [Gallus gallus] | | | 69 | | |  | | | 77.33/6.65 | | | |  |
| 240 | 1 | gi∣429836849 | | | Peroxiredoxin-1 [Gallus gallus] | | | 254 | | | n.a./n.a. | | | 22.53/8.24 | | | |  |
|  | 2 | gi∣71274075 | | | Ovotransferrin BB type [Gallus gallus] | | | 163 | | |  | | | 79.61/6.85 | | | |  |
|  | 3 | gi∣422920046 | | | Chain I, Structure Of The Anti-ptau Fab (pt231/ps235_1) In Complex With Phosphoepitope Pt231/ps235 | | | 122 | | |  | | | 22.43/5.13 | | | |  |
|  | 4 | gi∣126608 | | | RecName: Full=Lysozyme C; AltName: Full=Allergen Gal d Ⅳ; AltName: Allergen=Gal d 4; Flags: Precursor | | | 115 | | |  | | | 16.74/9.37 | | | |  |
|  | 5 | gi∣58332100 | | | Protease, serine, 2(trypsin 2) precursor [Xenopus (Silurana) topicalis] | | | 111 | | |  | | | 26.13/6.38 | | | |  |
|  | 6 | gi∣230359 | | | Chain A, Sturcture Of Triose Phosphate Isomerase From Chicken Muscle | | | 99 | | |  | | | 26.76/7.26 | | | |  |
|  | 7 | gi∣63052 | | | Unnamed protein product [Gallus gallus] | | | 80 | | |  | | | 17.69/5.35 | | | |  |
|  | 8 | gi∣326919110 | | | PREDUCTED: plasma kallikrein-like [Meleagris gallopavo] | | | 59 | | |  | | | 83.73/7.40 | | | |  |
|  | 9 | gi∣46048771 | | | Adenylate kinase isoenzyme 1 [Gallus gallus] | | | 55 | | |  | | | 21.78/8.68 | | | |  |
|  | 10 | gi∣449272815 | | | Alpha-2-macroglobulin-like protein 1, partial [Columba livia] | | | 53 | | |  | | | 159.69/6.39 | | | |  |
